# Gearing up for battle: Harnessing adaptive T cell immunity against gram-negative pneumonia

**DOI:** 10.3389/fcimb.2022.934671

**Published:** 2022-08-18

**Authors:** Catherine A. Gao, Luisa Morales-Nebreda, Chiagozie I. Pickens

**Affiliations:** Department of Medicine, Division of Pulmonary and Critical Care Medicine, Northwestern University Feinberg School of Medicine, Chicago, IL, United States

**Keywords:** gram-negative pneumonia, T cells, host – bacteria interaction, adaptive immunity

## Abstract

Pneumonia is one of the leading causes of morbidity and mortality worldwide and Gram-negative bacteria are a major cause of severe pneumonia. Despite advances in diagnosis and treatment, the rise of multidrug-resistant organisms and hypervirulent strains demonstrates that there will continue to be challenges with traditional treatment strategies using antibiotics. Hence, an alternative approach is to focus on the disease tolerance components that mediate immune resistance and enhance tissue resilience. Adaptive immunity plays a pivotal role in modulating these processes, thus affecting the incidence and severity of pneumonia. In this review, we focus on the adaptive T cell responses to pneumonia induced by *Klebsiella pneumoniae*, *Pseudomonas aeruginosa*, and *Acinetobacter baumannii*. We highlight key factors in these responses that have potential for therapeutic targeting, as well as the gaps in current knowledge to be focused on in future work.

## Introduction

Gram-negative bacteria are a leading cause of severe hospital-acquired pneumonia/ventilator-associated pneumonia (HAP/VAP), which can progress to acute respiratory distress syndrome (ARDS), multi-organ failure, and death (Jean et al., 2020). Despite advances in diagnosis and management of severe pneumonia, it remains associated with high rates of mortality, prolonged hospitalizations and substantial healthcare expenditures (Torres et al., 2021). A cohort of nosocomial pathogens with significant multi-drug resistant (MDR) and extensively drug resistant (XDR) characteristics are grouped together with the acronym, ESKAPE (*Enterococcus faecium*, *Staphylococcus aureus*, *Klebsiella pneumoniae*, *Acinetobacter baumannii*, *Pseudomonas aeruginosa*, and *Enterobacter* spp), due to their ability to ‘escape’ traditional antimicrobial therapy (Mulani et al., 2019). Four of these six hypervirulent pathogens are Gram-negative bacteria, underscoring the critical need to address gaps in the treatment of lower respiratory tract infection from these pathogens. Successful resolution of any severe infectious disease requires a multipronged approach to management, but work in severe pneumonia has primarily focused on developing therapeutics that target specific pathogens. Furthermore, even after receiving optimal antimicrobial therapy, mortality remains exceedingly high for patients with Gram-negative pneumonia (Feng et al., 2019). Thus, leveraging non-traditional strategies focusing on host-derived immune mechanisms that promote restoration of tissue architecture and physiological function are critical for improved patient outcomes and survival (Theuretzbacher and Piddock, 2019). Optimal therapies for severe pneumonia must address both pathogen and host, and a comprehensive characterization of the innate and adaptive immune response during severe pneumonia is a critical step toward achieving that goal.

T cells are a central component of protective immunity to severe pneumonia (Chen and Kolls, 2013). Based on cell surface expression markers and specialized function, T cells are divided into the classic dichotomy of CD4^+^ and CD8^+^ T cells. CD4^+^ T cells, also known as T Helper (T_H_) cells, support antibody-generating B cells and coordinate host defenses against pathogens through myriad functions. Based on their ability to express signature cytokines and lineage-specifying transcription factors, effector CD4^+^ T cells are divided into five classic subsets: T_H_1, T_H_2, T_H_17, T follicular helper (T_FH_) and regulatory T (Treg) cells (Annunziato and Romagnani, 2009). Optimal defense against acute viral and bacterial pneumonia requires IFN-γ-producing T_H_1 cells, whereas T_H_2 immunity confers protection from toxins, parasites, and in some cases, chronic bacterial infections (Wu et al., 2020). Context-dependent cues can lead to functional reprogramming and phenotypic plasticity of these subsets, which can often limit the ability to discern beneficial from pathogenic cells in a given disease condition (DuPage and Bluestone, 2016). Nonetheless, a detailed understanding of the molecular mechanisms that govern effector CD4^+^ T lineage heterogeneity and tissue-specific functional adaptability during an infectious process is crucial for developing new host-derived pharmacotherapies for treating pneumonia. Cytotoxic CD8^+^ T cells (CTLs) mediate direct killing of infected target cells. These T cells can be further subdivided into naive, effector, and memory subsets based on their activation status (Seder and Ahmed, 2003). Altogether, distinct T cell subsets orchestrate innate and adaptive immune responses, pathogen clearance, tissue repair, and establishment of long-lasting memory to respiratory bacterial infections (Chen and Kolls, 2013). Here, we identify similarities and differences in the adaptive T cell immune response to three common Gram-negative bacteria: *Klebsiella pneumoniae*, *Pseudomonas aeruginosa*, and *Acinetobacter baumannii*. We also highlight gaps in the current understanding of the host immune defenses to Gram-negative pneumonia, which could be exploited to develop the next generation of immunomodulatory small molecule- and T cell-based therapeutics to control lung inflammation.

### Klebsiella pneumoniae

*Klebsiella pneumoniae* (Kp) is a Gram-negative, encapsulated bacillus from the Enterobacteriaceae family. Kp is found primarily in the human gastrointestinal tract of a significant percent of the population, and in even higher proportions in hospitalized patients (Ashurst and Dawson, 2022). *Klebsiella* pneumonia carries mortality ranging from 25 -50% (Venkataraman et al., 2018) and *Klebsiella* species cause a significant number of HAP and VAP (Jones, 2010), more commonly than CAP. It also frequently causes bacteremia, which in turn leads to increased mortality, especially in those who are older, require mechanical ventilation, or have alcohol use disorder (Chetcuti Zammit et al., 2014).

Kp isolates have multiple virulence factors including a polysaccharide capsule that can evade phagocytosis, complement and antimicrobial molecules (Paczosa and Mecsas, 2016). Hypervirulent Kp isolates can acquire antimicrobial resistance through horizontal transfer of plasmids, leading to the formation of MDR Klebsiella strains— including ones with potent antibiotic resistance to extended-spectrum beta-lactamases (e.g., CTX-M and AmpC-type ESBLs) and carbapenemases (e.g., KPC, NDM, and VIM) (Pitout et al., 2015). Therapeutic options remain limited for these isolates and are often toxic; meanwhile, MDR prevalence continues to rise in hospitals worldwide, representing a major public health threat worldwide (Bassetti et al., 2018).

Numerous components of the innate immune system play an important role in the fight against Kp. Experimental models showed that alveolar macrophages, monocytes, and neutrophils contribute to the resolution of Kp infection (Xiong et al., 2015; Roger et al., 2013). Neutrophils appear to be the major initial defense against Kp, as demonstrated in murine models with dysregulated neutrophil function and chemotaxis (Hirche et al., 2005; Cai et al., 2010). The IL-17 family of cytokines is a critical mediator in the recruitment of neutrophils to the site of pulmonary Kp infection, and innate-like lymphocytes (i.e., γδ T cells) constitute one of the primary early sources of IL-17 (Price et al., 2012). γδ T cells are a low abundance and specialized subset of T cells, that accumulate in multiple peripheral tissues— including the lung— where they are key contributors to tissue homeostasis and immune surveillance during infection (Ribot et al., 2021). Indeed, mice genetically deficient in γδ T cells challenged with Kp exhibited decreased production of TNF-α and IFN-γ cytokines, higher peripheral bacterial dissemination and worsened survival when compared to wild-type or αβ T cell-deficient mice (Moore et al., 2000).

**Figure 1:**
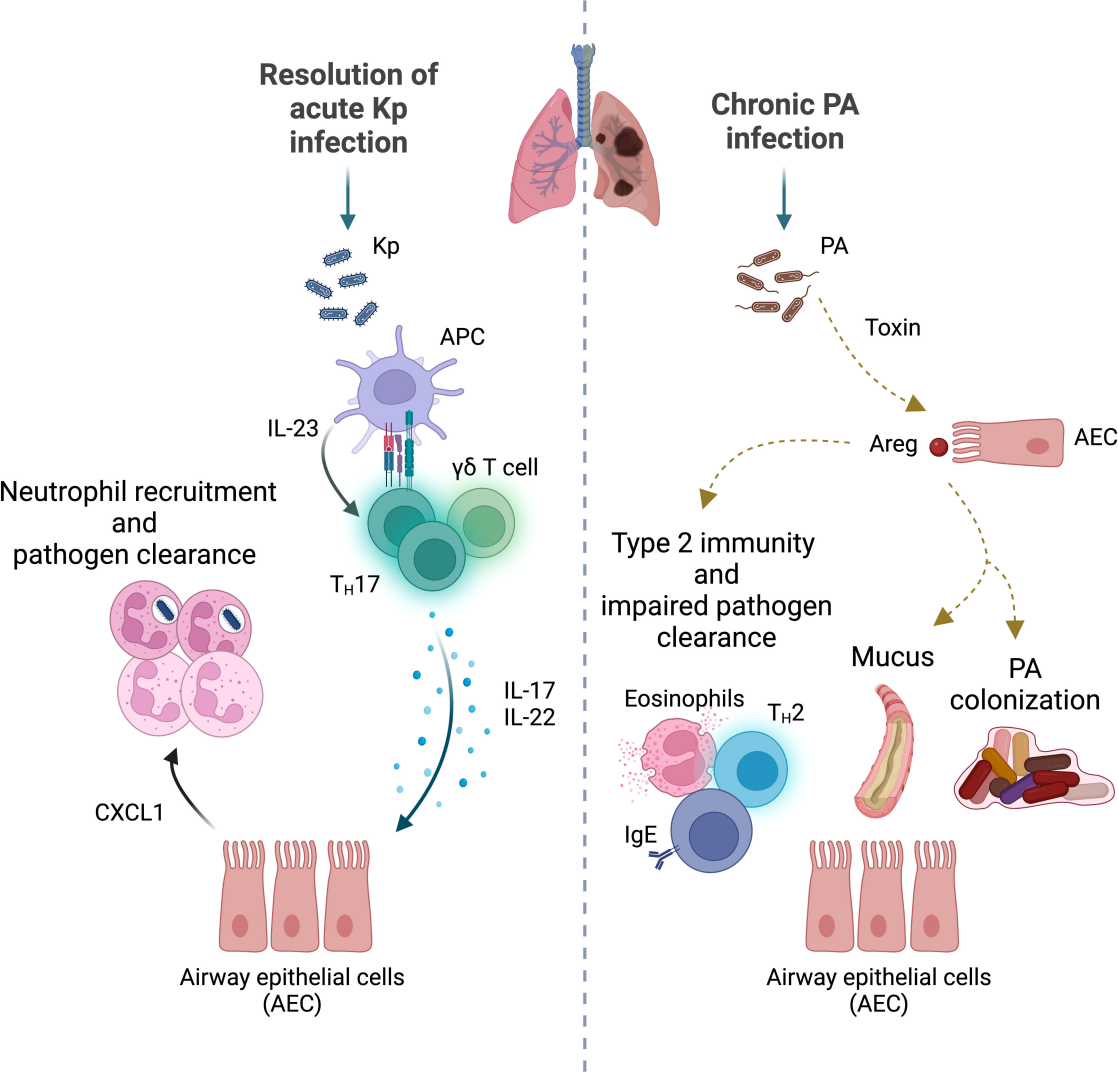
Protective versus pathogenic T cell immunity to Gram-negative bacteria. **Left panel:** Upon infection with *Klebsiella pneumoniae* (Kp), antigen presenting cells (APC) recognize and present pathogenic peptides to T cell subsets in local lymph nodes. APC release polarizing cytokines (e.g., IL-23) that activate T_H_17 and γδ T cells. These T cell subsets produce IL-17 and stimulate airway epithelial cells (AEC) to express chemokines (e.g., CXCL1) that mediate neutrophil recruitment to the infection site. Activated neutrophils phagocytose and kill bacteria, therefore enhancing Kp clearance and resolution of local inflammation. IL-22 serves a protective role by promoting AEC repair. **Right panel:** *Pseudomonas aeruginosa* (PA)-derived toxin activates the amphiregulin (Areg) – Epidermal Growth Factor (EGF) receptor pathway to induce a type 2 inflammatory response and increase mucus production, enhancing pathogen survival. Deviation of the immune response toward pathogenic expansion of T_H_2 immunity, creates a favorable niche for PA colonization and chronic infection. Chronic PA exposure can result in increased T_H_17 cell activation, persistent neutrophil recruitment, and lung tissue damage (*not shown*).

The effector CD4^+^ T cell lineage is a critical component of adaptive immunity against Gram-negative bacteria, as it is required for almost all adaptive immune defense mechanisms against these microbes. IL-17-producing CD4^+^ effector T (T_H_17) cells were initially described for their pathogenic role as major drivers of autoimmune inflammation (Harrington et al., 2005). Since their discovery, multiple studies have identified a protective role for T_H_17-mediated host immune responses against extracellular bacteria in the lung. Following intranasal delivery of Kp, *Il-17ra*-deficient mice exhibited decreased production of granulocyte colony-stimulating factor (G-CSF) and CXC-chemokine ligand 2 (CXCL2, also known as MIP-2) resulting in impaired granulopoiesis and neutrophil chemotaxis (Ye et al., 2001). Delayed recruitment of neutrophils to the lung led to a significant increase of Kp burden 24-hours post-infection and worsened mortality when compared to control animals. Following on these observations, Happel et al. went on to demonstrate that the IL-12 family member and pro-inflammatory cytokine, IL-23, is the dominant, upstream regulator of lung-specific IL-17 production following Kp challenge (Happel et al., 2005). *Il-23p19* subunit knockout (KO) mice showed decreased expression of multiple cytokines and chemokines known to be upregulated by IL-17, including G-CSF. Altered expression of these inflammatory mediators was exclusively restored through intratracheal inoculation of murine recombinant IL-17 to *Il-23p19* KO mice (Happel et al., 2005). Moreover, the T_H_17-derived and IL-23-regulated effector molecule, IL-22, was subsequently found to exert a protective function against Kp through modulation of alveolar epithelial repair (Aujla et al., 2008). Collectively, these studies support a key role for the T_H_17 cell lineage and effector IL-23/IL-17/IL-22 signaling pathways orchestrating host defense against Gram-negative bacterial infection in the lung **(** **Figure 1** **)**. Future studies should address the contribution that IL-17-specific cell origin, phase of bacterial infection (early or late), and distinctive T_H_17 phenotypic and effector programs have on lung host defense against Kp.

Establishment of immunological T cell memory is paramount for controlling pneumonia-susceptibility throughout the lifespan (Smith et al., 2018). Tissue-resident memory (T_RM_) cells are a unique T cell subpopulation that accumulates in peripheral tissues to enhance regional immunity and regulate recall responses to local pathogens. In a seminal study, Amezcua Vesely and colleagues used inducible, fate-mapping murine models and parabiosis experiments to demonstrate that lung CD4^+^ T_RM_ cells are partially derived from T_H_17 cells (Amezcua Vesely et al., 2019). Immunization with heat-killed Kp, resulted in the generation of T_H_17 cells and a long-lived “ex T_H_17” population that persisted in the lung as T_RM_ cells. Immunized and control mice were then infected with live MDR-Kp, with the former group exhibiting a protective phenotype when compared to the non-immunized (control) group. A subsequent elegant study by Iwanaga et al. found that immunization with a Kp vaccine formulated with the conserved outer membrane protein X (OmpX) and the T_H_17 adjuvant LTA-1, induced a lung-specific population of T_H_17 - T_RM_ cells that conferred heterotypic protection against multiple Kp serotypes in an adoptive cell transfer model (Iwanaga et al., 2021). The majority of available pneumonia vaccines target polysaccharide antigens that mostly unleash serotype-specific humoral immunity (Avci et al., 2011). Thus, these observations carry important implications in the development of novel cell-based immunotherapies and serotype-independent mucosal vaccines, that could elicit lung protective T_RM_ cells against MDR strains and phylogenetically-related Gram-negative bacteria of the Enterobacteriaceae family.

### Pseudomonas aeruginosa

*Pseudomonas aeruginosa* (PA) is a ubiquitous Gram-negative rod that has become one of the most common causes of HAP and VAP (Rello et al., 2002). While rarely a cause of CAP (Fine, 1996), PA VAP has high mortality rates, ranging from 42-87% (Fujitani et al., 2011) and is amongst the most burdensome of healthcare-associated infections due to its prevalence and pathogenicity (Lambert et al., 2011).

PA infection alters normal airway clearance through disruption of ciliary activity and injury to airway epithelium, resulting in increased mucus secretion (Sethi and Murphy, 2008). Furthermore, PA can form biofilm matrices (Ma et al., 2009), which protect the bacteria against environmental stresses and mitigate antibiotic efficacy. PA-acquired antimicrobial resistance is multifactorial, and includes upregulation of efflux pumps, toxins, β-lactamases, and decreased outer membrane permeability, resulting in MDR isolates (Sun et al., 2011). Through frequent exposure to antibiotics, MDR-PA is often selected for in chronically infected patients such as those with cystic fibrosis (CF), non-CF bronchiectasis, and chronic obstructive lung disease, making these strains extremely difficult to eradicate with limited therapeutic options (Murray et al., 2007). Persistent or recurrent infection with PA in these patient populations is accompanied by an accelerated decline in pulmonary function, leading to early death or lung transplantation. Thus, understanding the mechanisms regulating effector functions of host immune resistance are paramount to develop novel therapeutic strategies for managing PA-associated lung infection.

There are several innate immune pathways activated by PA’s pathogen and biofilm-associated molecular patterns— which are recognized by Toll-like receptors 4 (TLR4) and 5 (TLR5) to initiate protective responses in the lung (Lin and Kazmierczak, 2017). Lung-patrolling tissue-resident alveolar macrophages (TRAMs) are the first immune cell responders to PA infection. As gatekeepers of the respiratory system, TRAMs express a network of chemokines and cytokines that result in alveolar recruitment of neutrophils, natural killer cells, dendritic cells and T cells, to fine-tune the immune response to PA infection.

Multiple studies have found a protective role for adaptive immune responses in murine models of acute PA infection (Powderly et al., 1986; Nieuwenhuis et al., 2002; Koh et al., 2009). Specifically, mice deficient in mature T and B cells (*Rag2* KO mice) exhibited impaired bacterial clearance and worsened survival post-PA inoculation (Nieuwenhuis et al., 2002; Koh et al., 2009). Chronic PA infections in patients with CF or non-CF bronchiectasis have been mostly associated with induction of a T_H_2 program in the lung, particularly during exacerbations (Moser et al., 2000; Hartl et al., 2006; Tiringer et al., 2013; Quigley et al., 2015). Instead, patients with an upregulated T_H_1 immune response had better lung function and prognosis, underscoring a key role for dysregulated T_H_ cell polarization in modulating severity of PA-induced lung infection (Moser et al., 2000). Bacteria can use signaling molecules to monitor their population density and regulate gene expression in a process known as quorum sensing (QS). Investigators have reported that certain *N-acyl* homoserine lactone QS-signal molecules, suppress T_H_1 cytokines and T cell proliferation, further skewing the T_H_1/T_H_2 balance (Telford et al., 1998; Skindersoe et al., 2009) toward a T_H_1-deficient environment— rendering patients more susceptible to PA infection (Moser et al., 2000; Brazova et al., 2005; Parker et al., 2012). Additionally, Agaronyan and colleagues, recently reported that a PA-derived toxin can induce type 2 inflammatory responses in mice, leading to increased host mucin production that serves as a nutrient source for bacteria (Agaronyan et al., 2022). Using a human epithelial cell line and murine lung organ cultures, they characterized a mechanism whereby the PA metalloprotease, LasB, induces expression of type 2-associated genes through activation of the epithelial growth factor family member, amphiregulin (Areg). Deviation of the immune response led to remodeling of the infectious niche and altered the T_H_1/T_H_2 balance to promote chronic PA infection. Taken together, these observations emphasize the complexity of host-pathogen interactions during PA infection, where PA-associated virulence factors can induce effector responses that, while beneficial for their survival, can promote bacterial colonization and drive specific immunopathology in the host **(** **Figure 1** **)**.

A single-cytokine categorization of T_H_ cell subsets incompletely describes the heterogeneity and plasticity of adaptive immune responses (O’Shea and Paul, 2010). Some T_H_ cells share a closer developmental connection, and in complex inflammatory microenvironments with mixed polarizing cues can result in hybrid T_H_ signatures (Tortola et al., 2020). Indeed, investigators found that an enriched T_H_2-T_H_17 program in the bronchoalveolar lavage fluid (BALF) of CF patients preceded PA infection (Tiringer et al., 2013). Multiple groups confirmed these findings by reporting increased levels of IL-17 and IL-17^+^CD4^+^ T cells in sputum, BALF and draining lymph node specimens from CF patients with chronic PA-induced lung infection (McAllister et al., 2005; Decraene et al., 2010; Chan et al., 2013). Notably, Mulcahy et al. subsequently showed that elevated T_H_17 responses in peripheral blood inversely correlated with lung function in this patient population (Mulcahy et al., 2015). Hence, to fathom the molecular mechanisms underpinning the role of T_H_17 functional programs during PA infection, investigators turned to experimental murine models. Similar to the Kp pneumonia model, T_H_17 responses have been broadly linked to a protective phenotype following acute PA infection (Liu et al., 2011; Wu et al., 2012; Baker et al., 2019). Results from chronic PA infection studies have been more difficult to interpret. The lack of typical human CF clinical manifestations in mice with targeted *CFTR* gene alterations has tempered the enthusiasm to examine T_H_17 immunity in CF murine models. While neutralization of IL-17 in a CF mouse model demonstrated protection against lung inflammation from PA (Hsu et al., 2016), other investigators found that *Il-17ra*-deficient mice exhibited higher mortality post-infection (Bayes et al., 2016). Collectively, these results highlight the need for studies that examine context-dependent, cell-specific mechanisms controlling the interaction between bacterial and host immune resistance programs— and their temporal effect in driving lung inflammation, injury and repair in novel experimental models.

In humans and mice, a specialized subset of CD4^+^ T cells— known as Treg cells— are recruited to the alveolar space in response to injury and promote resolution of lung inflammation and tissue regeneration by exerting myriad reparative functions (Walter et al., 2018; Morales-Nebreda et al., 2021). In a large cohort of pediatric and young adult patients with CF and non-CF bronchiectasis, Hector et al. observed a significantly decreased number of peripheral and BALF Treg cells with impaired suppressive function, when compared to healthy control subjects (Hector et al., 2015). Notably, reduced Treg cells correlated with worsened lung function and chronic PA colonization. Furthermore, in an acute PA pneumonia model, *Cftr*-deficient (*Cftr* ^-/-^) mice showed decreased percentages of Treg cells when compared to control animals (*Cftr* ^+/+^ *). Cftr* ^-/-^mice developed chronic inflammation, which was mitigated by adoptive transfer of wild-type *Cftr* ^+/+^, but not by transfer of *Cftr* ^-/-^ Treg cells. While the mechanisms that underlie the impairment in Treg cell-mediated reparative function in this specific model remain unknown, these results support the emerging evidence that Treg cell-based immunotherapeutics can be leveraged to treat complex inflammatory conditions— including acute lung inflammation associated with pneumonia (Joudi et al., 2022).

### Acinetobacter baumannii

*Acinetobacter baumannii* (AB) is an aerobic, Gram-negative bacillus responsible for a significant number of HAP and VAPs (Hartzell et al., 2007). The mortality rate for AB infection ranges from 26% to 55% and increases with infection by MDR strains (Xiao et al., 2017). Similar to Kp and PA, AB has numerous mechanisms of antibiotic resistance that adds to the difficulty in effective treatment of the disease (Fournier and Richet, 2006), including beta-lactamases that can hydrolyze carbapenems (Singh et al., 2013). Porin channels that allow antibiotic entry into bacteria are decreased in number and size in AB, which also facilitates antibiotic resistance (Mussi et al., 2005). Other mechanisms include biofilm formation, nutrient acquisition, and escape from the complement cascade (Russo et al., 2010; Wang et al., 2014; Geisinger and Isberg, 2015). These mechanisms enhance the ability of AB to infect and persist in the lower respiratory tract, and in turn, a robust and targeted immune response ensues.

Neutrophils are paramount to the innate immune response to AB. In mouse models, intratracheal infection of neutropenic mice with AB led to the development of severe acute lung injury and increased mortality when compared to control mice (Joly-Guillou et al., 1997). Consistent with this finding, multiple studies have demonstrated respiratory failure, neutropenic fever, and high rates of mortality in neutropenic patients diagnosed with AB pneumonia (Nunley et al., 2010; Li et al., 2020). Along with neutrophils, macrophages play a key role in the response to AB infection. Mouse models demonstrate the ability of macrophages to phagocytose AB shortly after infection leading to prompt release of pro-inflammatory cytokines and recruitment of additional innate and adaptive immune cell subsets (Qiu et al., 2012).

A detailed understanding of the molecular and cellular mechanisms governing adaptive immune responses to AB infection remains an area for much needed research (García-Patiño et al., 2017). While innate and adaptive IL-17 signaling pathways have been shown to be critical in neutrophil recruitment and defense against acute Kp and PA-associated pneumonia in mice, its role against AB infection is not clearly characterized (Yan et al., 2016). Using a systemic infection murine model of AB-induced sepsis, Breslow and colleagues demonstrated that IL-17-mediated immunity does not play a crucial role in controlling susceptibility to AB infection (Breslow et al., 2011). Specifically, they showed that AB infection induced biphasic increases in both IL-17 and the neutrophil keratinocyte-derived chemoattractant (KC/CXCL1), however administration of specific antibodies targeting these molecules, resulted in no difference in bacterial burden and mortality when compared to control conditions. Furthermore, *Il17a*-deficient mice inoculated with five distinct AB strains of different virulence level and antibiotic resistance pattern, demonstrated no difference in susceptibility when compared to wild-type mice (Breslow et al., 2011). IL-17A shares 50% homology of its amino acid sequence with the IL-17 family member, IL17F. Although they bind the same receptor, their context-dependent functional differences in modulating inflammatory conditions is an area of ongoing investigation (Tang et al., 2018). Hence, a compensatory role for IL-17F during AB infection remains a plausible explanation for the phenotype observed in the Breslow et al. report. Future work, leveraging *Il-17f-* and *Il-17ra*-deficient mice could better address the specific role of IL-17-dependent mucosal immunity in the susceptibility to AB-induced pneumonia.

Given the rising prevalence and mortality associated with MDR-and XDR-AB strains, particularly in immunocompromised hosts— alternative preventative strategies with vaccination of at-risk populations remain a promising avenue for investigation and immunotherapy development. An outer membrane-porin-based (rOmpA) vaccine was previously shown to protect mice against lethal infection from XDR-AB isolates (Luo et al., 2012). Following on these observations, Lin et al. went on to demonstrate that increased doses of a rOmpA-based vaccine resulted in an enhanced T_H_2 immune response in mice. T cell epitope mapping uncovered expansion of IL-4-producing T cells to a broader range of immunodominant peptides— suggesting epitope spreading— whereas only a restricted number of epitopes induced T_H_1 cells (Lin et al., 2013). Altogether, these studies underscore the substantial gap in our understanding of how distinct T cell subsets specifically contribute to host immune defenses against AB infection. Nonetheless, they provide a foundation from which future studies can be designed to elucidate the contribution of distinct T cell subsets to mucosal host immunity in the lung.

## Concluding remarks

Adaptive T cell immunity is central to the host defense against pulmonary infection by Gram-negative bacteria. Indeed, successful resolution of pneumonia requires generation of the appropriate effector T cell response to any given pathogen (Sallusto, 2016). As pathogens modify infectious niches within the host, T cells polarize their functional programs to adapt to a changing microenvironment. Reshaping of T cell responses can be either beneficial to the host, through microbe eradication and promotion of tissue repair, or detrimental by driving immunopathology and bacterial colonization. Thus, understanding the complex circuitry of regulatory mechanisms that control host immune T cell resistance pathways throughout the course of pneumonia can be exploited to generate new therapies that amplify T cell-mediated immune resistance and tissue resilience in the host. The multitude of resistance mechanisms and virulence factors associated with *Klebsiella pneumoniae*, *Pseudomonas aeruginosa*, and *Acinetobacter baumannii* have drastically narrowed the armamentarium of traditional pathogen-directed therapies for pneumonia, and rendered most antibiotics ineffective (Pickens and Wunderink, 2019). Hence, non-traditional, host-specific approaches are needed to circumvent bacterial drug resistance to effectively manage pneumonia. The development of cutting-edge technologies in the fields of synthetic immunology, genome and epigenome editing systems has broadened the capacity to engineer T cells that can either impart specificity against a given microbe or pathogenic immune cell, or expand tissue-protective T cell subsets (Ferreira et al., 2019). The COVID-19 pandemic has renewed the interest to leverage existing platforms such as viral vectored- or nucleic-acid-based vaccines to achieve sterilizing mucosal immunity against bacterial infections. Recently, a group of investigators showed the feasibility to produce *in vivo* engineered T cells, by injecting T cell-targeted lipid nanoparticles with modified mRNA to ameliorate cardiac fibrosis in mice (Rurik et al., 2022). A growing body of preclinical data supports the use of these combinatorial approaches to treat pathologies beyond cancer, including autoimmune, metabolic and infectious diseases (Aghajanian et al., 2022). Thus, we envision that coupling of these technologies holds tremendous promise in harnessing the next generation of T cell-based immunomodulatory pharmacotherapies to treat an expanding population of patients with severe Gram-negative pneumonia.

## Author contributions

All authors contributed to the article and approved the submitted version.

## Funding

CG is supported by NIH NHLBI Training Grant T32HL076139. LM-N is supported by NIH award K08HL1593356 and the Francis Family Foundation’s Parker B. Francis Opportunity Award. CP is supported by NIH/NIAID U19AI135964.The content is solely the responsibility of the authors and does not necessarily represent the official views of the funding sources.

## Conflict of interest

The authors declare that the research was conducted in the absence of any commercial or financial relationships that could be construed as a potential conflict of interest.

## Publisher’s note

All claims expressed in this article are solely those of the authors and do not necessarily represent those of their affiliated organizations, or those of the publisher, the editors and the reviewers. Any product that may be evaluated in this article, or claim that may be made by its manufacturer, is not guaranteed or endorsed by the publisher.
